# Efficacy prediction of systemic immune-inflammation index and prognostic nutritional index in breast cancer patients and their variations after neoadjuvant chemotherapy

**DOI:** 10.3389/fimmu.2025.1514736

**Published:** 2025-05-09

**Authors:** Jingyi Ni, Xue Qi, Conghui Jin, Weiwei Xu, Xinghui Li, Li Song, Xunlei Zhang

**Affiliations:** ^1^ Department of Oncology, Affiliated Tumor Hospital of Nantong University, Nantong, Jiangsu, China; ^2^ Department of Oncology, Nantong Liangchun Hospital of Traditional Chinese Medicine, Nantong, Jiangsu, China; ^3^ Department of Breast Surgery, Affiliated Tumor Hospital of Nantong University, Nantong, Jiangsu, China

**Keywords:** breast cancer, systemic immune-inflammation index, prognostic nutritional index, neoadjuvant chemotherapy, prognosis

## Abstract

**Objective:**

The purpose of this study was to evaluate the predictive values of systemic immune-inflammatory index (SII), prognostic nutrition index (PNI), change of SII (ΔSII), change of PNI (ΔPNI) and ΔPNI-ΔSII score in patients with neoadjuvant chemotherapy for breast cancer.

**Methods:**

We enrolled in a retrospective study involving 72 patients with breast cancer between February 2020 and January 2022. All patients had clinical features of axillary lymph node metastasis and received neoadjuvant therapy. PNI and SII were detected by hematology before and after treatment. Chi-square test was used to compare the clinicopathological and experimental parameters among all groups. Logistic regression analysis was used to evaluate the prognostic value of each factor.

**Results:**

The prognosis was evaluated and 18 patients (25%) achieved pathological complete response (pCR) after neoadjuvant therapy. The pCR rate of breast cancer patients was significantly correlated with ER, PR, HER-2, molecular subsets, tumor size, vascular invasion, nerve invasion, N stage, clinical stage and chemotherapy regimen. Low ΔPNI, high ΔSII and higher ΔPNI-ΔSII score values had better prediction of therapeutic effect, especially the ΔPNI-ΔSII score.

**Conclusion:**

In breast cancer patients receiving neoadjuvant chemotherapy, ΔPNI-ΔSII score is an effective predictor of efficacy, which helps to identify high-risk groups and evaluate efficacy.

## Introduction

Breast cancer, one of the most common cancers among women worldwide, is also the leading cause of cancer-related death among women worldwide (1). Breast cancer accounts for 30 percent of newly diagnosed cancers in women and 15 percent of cancer deaths in women, according to available data (2). According to international guidelines, it is treated in a variety of ways, including surgery, chemotherapy, targeted therapy, hormone therapy, radiation therapy and immunotherapy (2). The reality is that surgery is not an optimal option, especially for breast cancer patients with axillary lymph node metastases. Neoadjuvant chemotherapy is a systemic therapy before local treatment methods. Preoperative neoadjuvant chemotherapy is becoming more and more important in breast cancer patients, with potential benefits such as reducing clinical period, improving breast preservation rate and reducing distant spread (3, 4). However, not all breast cancer patients achieve complete remission after receiving neoadjuvant chemotherapy (2). Clinical, pathological and molecular indicators can be generally used in predicting therapeutic effect at present. There is still a lack of dependable indicators to predict tumor response and efficacy in patients before individualized treatment.

In recent years, tumor microenvironment and nutrition have been paid more and more attention in the prognosis of tumor patients (5). A variety of inflammatory cells and mediators are important components of tumor microenvironment. Current evidence suggests that inflammation and immunity play a crucial role in the development, progression of tumors, and therefore affect the effectiveness of treatment. Circulating inflammatory and immune cells involve neutrophil, lymphocyte, monocyte and platelet in peripheral blood (6). The systemic immunoinflammatory index (SII) is a novel inflammatory index, which is calculated based on peripheral blood (6). High SII is considered to be an independent negative prognostic in cancer patients. The prognostic nutrition index (PNI) is a novel nutrition index based on the serum albumin concentration and peripheral blood lymphocyte count (7). According to the recent studies, PNI has prognostic value in multiple cancer types (7–9). Some studies have shown that the lower the PNI of cancer patients, the worse the survival rate (10).

At present, it is mentioned above that immune inflammation and nutritional status have an impact on the prognosis of breast cancer, and SII and PNI could be effective predictive factors. However, there are many factors affecting nutritional and immune status, leading to the low correlation between SII/PNI and neoadjuvant chemotherapy efficacy. We assumed that ΔPNI and ΔSII, indicating that changes in nutritional and immune status before and after neoadjuvant chemotherapy, can better reflect the patients’ response to chemotherapy drugs. As no relevant literature to explore the predictive efficiency of ΔPNI and ΔSII for neoadjuvant chemotherapy response, we firstly conducted this study to determine the best parameters for predicting treatment sensitivity in breast cancer patients using a new marker, the ΔSII-ΔPNI score. The ΔSII-ΔPNI score represents a combination of inflammation and nutritional status changes before and after neoadjuvant chemotherapy.

## Materials and methods

### Patients

In this study, 72 patients with breast cancer in Affiliated Tumor Hospital of Nantong University were retrospectively analyzed. All cases was confirmed dependent on histological examination between February 2020 and January 2022. The inclusion criteria were: female breast cancer with histologically confirmed invasive breast cancer with axillary lymph node metastasis, which had complete clinical information, laboratory data. This study was approved by the Ethic Committee of Affiliated Tumor Hospital of Nantong University. All data used in this study are in accordance with the principles of the Declaration of Helsinki. Informed consent was not required for this retrospective study.

### Data collection

Patient clinicopathological information including age, full blood counts, routine biochemical examination, tumor size, differentiation, vascular invasion, nerve invasion, lymph node metastasis, TNM stage and details of treatment types were obtained from the medical records. All blood samples were collected within one week before neoadjuvant therapy and surgery, and tested on the machine within 48 hours. The inspection result data were directly obtained from the inspection report system. The collection, calculation, and analysis of data were completed by two doctors independently. Blood biomarkers include the platelet (P) counts, neutrophil (N) counts, lymphocyte (L) counts, albumin (Alb) levels. The PNI and SII were calculated as follows: PNI = Alb (g/L)+5×L(10^9^/L) (11), SII = P×[N/L] (12), ΔPNI = pre-therapy PNI - post-therapy PNI, ΔSII = pre-therapy SII - post-therapy SII. Some breast patients achieved pathologic complete response (pCR) after neoadjuvant chemotherapy, in which no tumor was found on pathologic examination after surgical resection.

### Treatment

Patients in this study all received 2–8 cycles of neoadjuvant chemotherapy based on paclitaxel, Trastuzumab and Pertuzumab for human epidermal growth factor receptor-2 (Her-2) positive tumor. The types of chemotherapy for TNBC, luminal A and luminal B subtypes included TAC regimen (Taxotere + Adriamycin + Cyclophosphamide), AT regimen (Adriamycin + Taxol) and AC-T regimen (Adriamycin + Cyclophosphamide follow Taxol). All patients underwent radical mastectomy after neoadjuvant chemotherapy.

### Statistical analyses

SPSS 23.0 software was used for statistical analyses. The relationship between the clinicopathological and laboratory parameters were evaluated using the chi-square test. Logistic regression analysis was used to evaluate the factors affecting the therapeutic effect of breast patients. Variables with *p* value <0.05 were enrolled in multivariate Cox hazard regression analysis to predictive factors for OS. *P-* value <0.05 was considered statistically significant.

## Result

### Baseline characteristics of patients

In this study, 72 patients with axillary lymph node metastasis of breast cancer were included in a retrospective analysis. **Table 1** summarized the clinical characteristics of breast cancer patients. All patients were female, with a median age of 53.5 years old, ranging from 21 to 74. The molecular subgroups were Lumina A in 12 cases (16.67%), Lumina B in 24 cases (33.33%), Her-2 positive in 23 cases (31.94%), and Triple negative in 13 cases (18.06%). 18 cases (25%) achieved pCR after neoadjuvant chemotherapy. The pre-treatment PNI and SII ranged from 40.05 to 67.7 and 203.29 to 2981.42, respectively. The median values of pre-treatment PNI and SII were 53.675 and 674.39. As demonstrated in **Table 1**, the pCR rate of breast patients was significantly correlated with ER, PR, HER-2, molecular subgroup, tumor size, vascular invasion, nerve invasion, N stage, clinical stage and chemotherapy regimen.

**Table 1:** Patient information and tumor characteristics.

Characteristics	Case(%)	pCR		P	PNI		P	SII		P
		Yes	No		<55.5	≥55.5		<929	≥929	
Age				0.881			0.753			0.499
<60	51(70.83)	13	38		32	19		38	13	
≥60	21(29.17)	5	16		14	7		14	7	
ER				<0.001			1.000			0.599
Negative	36(50.00)	16	20		23	13		27	9	
Positive	36(50.00)	2	34		23	13		25	11	
PR				0.011			0.753			0.630
Negative	51(70.83)	17	34		32	19		36	15	
Positive	21(29.17)	1	20		14	7		16	5	
HER-2				<0.001			0.371			0.745
Negative	41(56.94)	2	39		28	13		29	12	
Positive	31(43.06)	16	15		18	13		23	8	
Ki-67				0.491			0.662			0.068
<14	7(9.72)	1	6		5	2		3	4	
≥14	65(90.28)	17	48		41	24		49	16	
Molecular subgroup				<0.001			0.099			0.898
Luminal A	12(16.67)	0	12		11	1		9	3	
Luminal B	24(33.33)	2	22		12	12		16	8	
HER-2 positive	23(31.94)	14	9		14	9		17	6	
Triple negative	13(18.06)	2	11		9	4		10	3	
Location				0.557			0.655			0.905
Right	44(61.11)	12	32		29	15		32	12	
Left	28(38.89)	6	22		17	11		20	8	
Tumor size(cm)				<0.001			0.107			0.040
≤2	44(61.11)	18	26		28	16		33	11	
2< and ≤5	18(25.00)	0	18		9	9		15	3	
>5	10(13.89)	0	10		9	1		4	6	
Vascular invasion				<0.001			0.792			0.612
None	43(59.72)	18	25		28	15		32	11	
Yes	29(40.28)	0	29		18	11		20	9	
Nerve invasion				0.015			0.144			0.914
None	57(79.17)	18	39		34	23		41	16	
Yes	15(20.83)	0	15		12	3		11	4	
N stage				<0.001			0.971			0.515
N0	32(44.44)	18	14		20	12		24	8	
N1	25(34.72)	0	25		16	9		17	8	
N2	4(5.56)	0	4		3	1		4	0	
N3	11(15.28)	0	11		7	4		7	4	
Clinical stage				<0.001			0.009			0.635
0	18(25.00)	18	0		8	10		15	3	
I	11(15.28)	0	11		11	0		8	3	
II	23(31.94)	0	23		12	11		16	7	
III	20(27.78)	0	20		15	5		13	7	
Chemotherapy regimen				<0.001						0.747
TCbHP	30(41.67)	16	14		18	12	0.790	23	7	
TAC	38(52.78)	2	36		25	13		26	12	
Others	4(5.56)	0	4		3	1		3	1	
Chemotherapy cycles				0.818			0.244			0.961
<6	7(9.72)	2	5		3	4		5	2	
≥6	65(90.28)	16	49		43	22		47	18	

The bold P values denote statistical significance (P < 0.05).

### Efficacy prediction evaluation of PNI, SII, ΔPNI, ΔSII and ΔPNI-ΔSII in breast cancer

**Figure 1:**
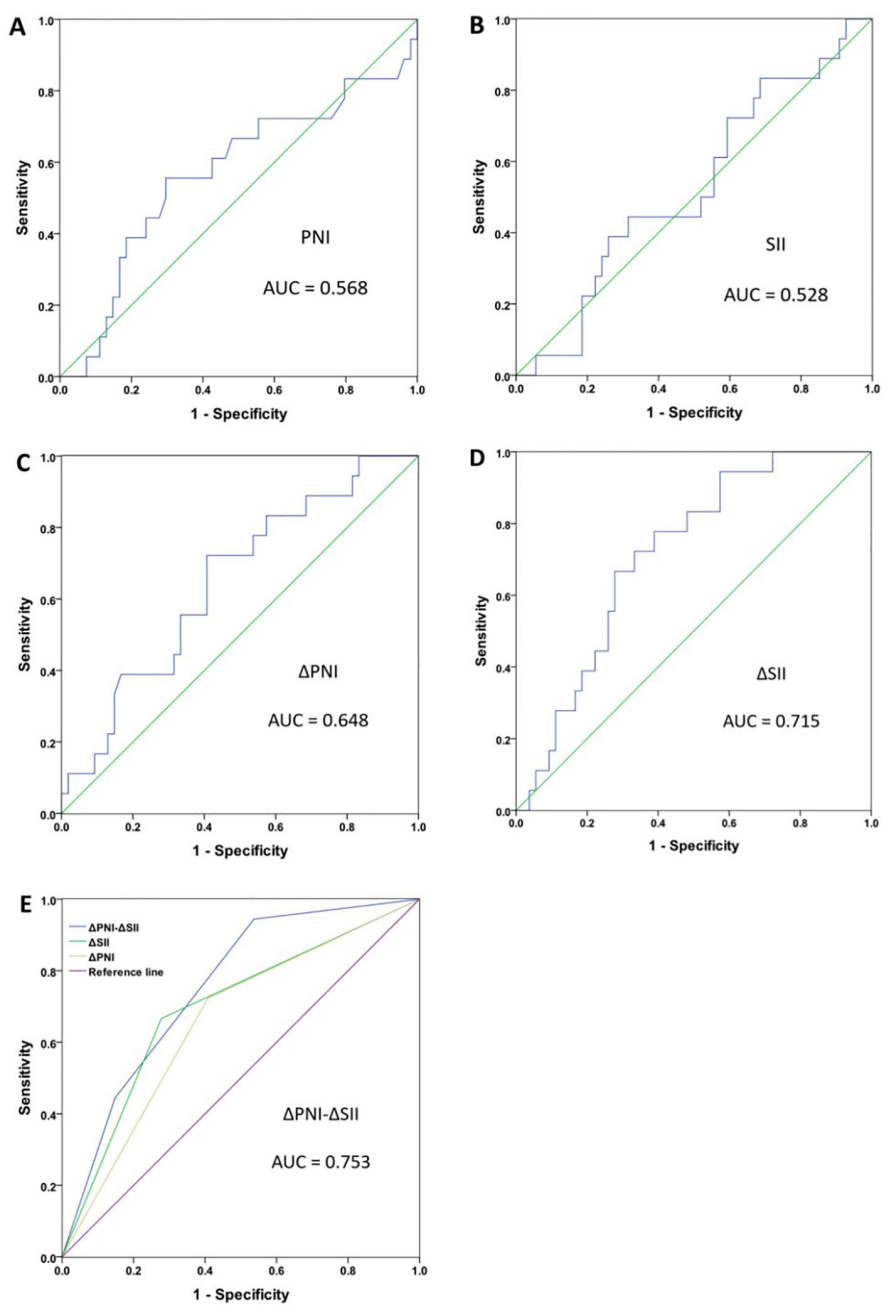
Receiver operating characteristic curve (ROC) analyses for breast cancer patient. PNI **(A)**, SII **(B)**, ΔPNI **(C)**, ΔSII **(D)**, ΔPNI-ΔSII **(E)**.

We constructed the ROC curve and calculated the AUC values to assess the predictive ability of SII, PNI, ΔPNI, ΔSII and ΔPNI-ΔSII to determine the cut-off value. The AUC values for these indicators were as follows: PNI AUC=0.568 (95%CI 0.404-0.733, p=0.387), SII AUC=0.528 (95%CI 0.378-0.478, p=0.725), ΔPNI AUC=0.648 (95%CI 0.507-0.789, p=0.062), ΔSII AUC=0.715 (95%CI 0.593-0.838, p=0.007) (**Figures 1A–D**).

Furthermore, the optimal cut-off value was 55.5 for PNI, 929 for SII, 3.925 for ΔPNI, and 169.153 for ΔSII. According to these results, patients with ΔPNI ≥3.925 and ΔSII <169.153 were assigned a ΔPNI-ΔSII of 0, patients with ΔPNI <3.925 or ΔSII ≥169.153 were assigned a ΔPNI-ΔSII of 1, and patients with ΔPNI <3.925 and ΔSII ≥169.153 were assigned a ΔPNI-ΔSII of 2. The AUC for ΔPNI-ΔSII was 0.753 (95%CI 0.612-0.836, p=0.002) (**Figure 1E**), indicating that the higher the value of ΔPNI-ΔSII, the better the prediction of treatment effect.

The low AUC values of PNI and SII indicate that the PNI and SII values before neoadjuvant therapy alone cannot reflect the effectiveness of neoadjuvant chemotherapy. A possible explanation is that there are many factors affecting nutritional and immune status alone, leading to the low correlation with neoadjuvant chemotherapy. The AUC values of ΔPNI and ΔSII are relatively higher, indicating that changes in nutritional and immune status before and after neoadjuvant chemotherapy can better reflect the patients’ response to chemotherapy drugs. The highest AUC value of ΔPNI-ΔSII may be explained by its combination of nutritional and immune changes, which can better reflect the patients’ nutritional reserve, immune status and physical condition, predict the patients’ tolerance and sensitivity to chemotherapy drugs, and thus better reflect the efficacy of neoadjuvant chemotherapy.

### Relationship between PNI, SII, ΔPNI, ΔSII, ΔPNI-ΔSII score with the clinicopathological parameters

According to the optimal threshold, 46 patients with PNI <55.55 were divided into low PNI group, 26 patients with PNI ≥55.55 were high PNI group, 52 patients with SII <929 were low SII group, and 20 patients with SII ≥929 were high ΔSII group. 35 patients with ΔPNI <3.925 were in the low ΔPNI group, 37 patients with ΔPNI ≥3.925 were in the high ΔPNI group, 45 patients with ΔSII <169.153 were in the low ΔSII group, and 27 patients with ΔSII ≥169.153 were in the high ΔSII group. PNI was correlated with clinical stage, SII was correlated with tumor size, as shown in **Table 1**. Patients with low ΔSII were negative for Her-2 (p<0.001)(**Table 2**). Analysis showed that ΔPNI-ΔSII score was significantly related to Her-2, tumor size and chemotherapy regimen.

**Table 2:** Relationship between ΔPNI, ΔSII, ΔPNI-ΔSII score with the clinicopathological parameters.

Characteristics	ΔPNI		P	ΔSII		P	ΔPNI-ΔSII			P
	<3.925	≥3.925		<169.153	≥169.153		0	1	2	
Age			0.681			0.639				0.799
<60	24	27		31	20		19	20	12	
≥60	11	10		14	7		7	10	4	
ER			0.814			0.224				0.817
Negative	17	19		20	16		12	15	9	
Positive	18	18		25	11		14	14	7	
PR			0.681			0.124				0.741
Negative	24	27		29	22		17	22	12	
Positive	11	10		16	5		9	8	4	
HER-2			0.658			<0.001				0.009
Negative	19	22		34	7		19	18	4	
Positive	16	15		11	20		7	12	12	
Ki-67			0.749			0.704				0.852
<14	3	4		5	2		3	3	1	
≥14	32	33		40	25		23	27	15	
Molecular subgroup			0.159			0.016				0.125
Luminal A	9	3		10	2		3	7	2	
Luminal B	9	15		15	9		11	8	5	
HER-2 positive	12	11		9	14		6	8	9	
Triple negative	5	8		11	2		6	7	0	
Location			0.436			0.803				0.420
Right	23	21		28	16		14	21	9	
Left	12	16		17	11		12	9	7	
Tumor size(cm)			0.315			0.005				0.037
≤2	24	20		22	22		12	18	14	
2< and ≤5	6	12		17	1		11	7	0	
>5	5	5		6	4		3	5	2	
Vascular invasion			0.598			0.154				0.187
None	22	21		24	19		12	21	10	
Yes	13	16		21	8		14	9	6	
Nerve invasion			0.321			0.116				0.068
None	26	31		33	24		22	20	15	
Yes	9	6		12	3		4	10	1	
N stage			0.240			0.052				0.373
N0	17	15		15	17		8	14	10	
N1	12	13		17	8		10	10	5	
N2	0	4		3	1		3	1	0	
N3	6	5		10	1		5	5	1	
Clinical stage			0.132			0.027				0.060
0	13	5		6	12		1	9	8	
I	4	7		7	4		5	4	2	
II	9	14		17	6		11	9	3	
III	9	11		15	5		9	8	3	
Chemotherapy regimen			0.779			<0.001				0.024
TCbHP	16	14		10	20		6	12	12	
TAC	17	21		31	7		18	16	4	
Others	2	2		4	0		2	2	0	
Chemotherapy cycles			0.436			0.803				0.420
<6	23	21		28	16		14	21	9	
≥6	12	16		17	11		12	9	7	

The bold P values denote statistical significance (P < 0.05).

### Efficacy evaluation significance of PNI, SII, ΔPNI, ΔSII, ΔPNI-ΔSII score in breast cancer patients

As shown in **Table 3**, patients who achieved pCR after treatment were significantly correlated with PNI (p=0.047), ΔPNI (p=0.021), ΔSII (p=0.003), ΔPNI-ΔSII (p=0.003). High PNI, low ΔPNI, high ΔSII and high ΔPNI-ΔSII were associated with higher pCR rate, and the SII before neoadjuvant chemotherapy had no significant with pCR rate (p=0.224). In addition, patients with higher ΔPNI-ΔSII score had better therapeutic effect. Logistic analysis (**Table 4**) showed that low ΔPNI increased the pCR rate of 2.782 times compared with the high value (p=0.025, 95%CI 1.179-12.131), and the high ΔSII increased the pCR rate of 4.2 times (p=0.05, 95%CI 1.652-16.369). Furthermore, higher ΔPNI-ΔSII had more pCR rate (p=0.01, 95%CI 1.699-9.342).

**Table 3:** Relationship between PNI, SII, ΔPNI, ΔSII, ΔPNI-ΔSII score with therapeutic effect.

Characteristics	pCR		P value
	Yes	No	
PNI			0.047
<55.5	8	38	
≥55.5	10	16	
SII			0.224
<929	15	37	
≥929	3	17	
ΔPNI			0.021
<3.925	13	22	
≥3.925	5	32	
ΔSII			0.003
<169.153	6	39	
≥169.153	12	15	
ΔPNI-ΔSII			0.003
0	1	25	
1	9	21	
2	8	8	

The bold P values denote statistical significance (P < 0.05).

**Table 4:** Logistic analyses of the PNI, SII, ΔPNI, ΔSII, ΔPNI-ΔSII score for the prediction of pCR rate in breast cancer patients.

Characteristics	B	SE	Wald	P value	OR (95% CI)
PNI (<55.5 vs. ≥55.5)	1.088	0.56	3.773	0.052	2.969 (0.990-8.900)
SII (<929 vs. ≥929)	-0.832	0.697	1.424	0.233	0.435 (0.111-1.706)
ΔPNI (≥3.925 vs. <3.925)	1.33	0.595	5.004	0.025	3.782 (1.179-12.131)
ΔSII (<169.153 vs. ≥169.153)	1.649	0.585	7.94	0.005	5.200 (1.652-16.369)
ΔPNI-ΔSII (0 vs. 1 vs. 2)	1.382	0.435	10.105	0.001	3.984 (1.699-9.342)

## Discussion

Nowadays, the treatment methods for breast cancer include surgery, chemotherapy, endocrine therapy for HR positive patients, targeted therapy for those Her-2 positive patients, radiotherapy, and immunotherapy for those triple-negative breast cancer (TNBC). Neoadjuvant chemotherapy plays an increasingly significant role in the treatment of breast cancer with axillary lymph node metastasis, but not all neoadjuvant chemotherapy has achieved the expected effect. Therefore, finding accurate predictors is very important for selecting the best treatment plan and improving the clinical efficacy of breast cancer patients.

Previous studies have explored the correlation between nutritional status and occurrence and development of malignant tumors (13–15). It is harmful for the survival and recovery of malnourished cancer patients. PNI is a nutritional evaluation index calculated by peripheral albumin and lymphocyte counts. Serum albumin can evaluate the long-term nutritional status (16, 17). Previous studies have confirmed that inflammatory response plays an important role in the initiation, invasive growth and metastasis of carcinoma (18).Therefore, the inflammatory still have important affecting in tumor progression (19). SII is a novel systemic immune-inflammation indicator which directly reflects the overall inflammatory and immune status of the patients (6, 20). Previous research explored that SII can predict prognosis of various carcinomas (21, 22). PNI and its variations had been proven to predict prognosis of breast cancer and lung cancer (23, 24). SII and its variations also had prognosis prediction in gastric or gastroesophageal junction cancer and non-small-cell lung cancer (25, 26). However, there is no relevant literature to study the correlation between ΔSII and breast cancer. Chung et al. conducted a retrospective study and showed that the changes in NLR (neutrophil-to-lymphocyte ratio) and PLR (platelet-to-lymphocyte ratio) can be used as predictors of neoadjuvant chemotherapy efficacy in TNBC. They also developed a prediction model, using a nomogram combine integrating blood tests (change NLR and PLR) and pre-treatment ultrasound parameter (presence of echogenic halo and H/W ratio), predicting pCR rate in TNBC receiving NAC (27). Another study explored the value of hemoglobin-albumin-lymphocyte-platelet (HALP) score, consists of four laboratory parameters including both nutritional and inflammatory status. Unfortunately, the results showed that both HALP and ΔHALP score have no significant association with neoadjuvant chemotherapy responses in breast cancer (28). In our study, we found that SII, PNI and their variations significantly predictive efficacy in breast cancer with neoadjuvant chemotherapy. Although some studies have found that SII and PNI are respectively related to the prognosis of breast cancer patients, no study has combined the two for correlation analysis. This study firstly investigated the efficacy prediction significance of the ΔSII-ΔPNI score in breast cancer patients. We hope to establish ΔSII-ΔPNI score to predict tumor response and therapeutic effect in patients with axillary lymph after neoadjuvant chemotherapy.

We found that the pCR rate of breast cancer patients was significant correlated with ER, PR, HER-2, molecular subsets, tumor size, vascular invasion, nerve invasion, N stage, clinical stage and chemotherapy regimen. Patients with high ΔSII, low ΔPNI and high ΔPNI-ΔSII score experienced better therapeutic effect. Patients with low ΔPNI means a certain nutritional status can get better curative effect. Li (22) et al. investigated the prognostic significance of the pre-treatment SII. However, in our study, we found that pre-treatment SII showed low AUC score (AUC=0.528), and ΔSII (AUC=0.715) may have better predictive reliability than pre-treatment SII. It is of great significance to find how to reduce the SII index. The change of SII attributed to the alterative of neutrophil, platelet, and lymphocyte counts. Neutrophils can secret a variety of inflammatory mediators to exert tumor-promoting activity (29). Platelets can prevent natural killer cells from dissolving cancer cells (30). Furthermore, lymphocytes can inhibit cancer progression through participating in cancer immune-surveillance (31). Lymphocytes are important components of the immune microenvironment, especially tumor-infiltrating lymphocytes, which are a mixture of CD8+ and CD4+ cells (32). Tumor-infiltrating CD8+ and CD4+ T lymphocytes inhibit cancer cell proliferation by inducing cancer cells apoptosis (33). Therefore, lymphocytes play key roles in anti-tumor immune reactions and tumor immune monitoring (34). High lymphocyte count can promote immunological responses in tumor microenvironment and inhibit cancer progression. During the neoadjuvant treatment of breast cancer, we can dynamically monitor ΔPNI-ΔSII score to guide the adjustment of the treatment strategies. For example, when the ΔPNI-ΔSII score is low, we can improve the patient’s nutritional status by strengthening nutritional support, supplementing high-quality protein. On the other hand, we can take measures to reduce local inflammation and increase the number of tumor infiltrating lymphocytes. Appropriate use of drugs targeting inflammatory pathways can also be considered. For example, anti-inflammatory drugs: COX-2 inhibitors (such as celecoxib) can inhibit prostaglandin synthesis and reduce inflammation driven tumor growth (35). Immunocheckpoint inhibitor: PD-1/PD-L1 inhibitor combined with anti-inflammatory treatment can reverse the immunosuppressive microenvironment and show efficacy in triple negative breast cancer (36). Targeted cytokines: IL-6 receptor antagonists (such as tropizumab) are explored for their effects on metastatic breast cancer in clinical trials (37). In summary, improving nutrition and immune-inflammatory environment can effectively improve the therapeutic effect.

There are several limitations to the study that need to be addressed. First of all, this study is a retrospective study, which may have some selection bias and cannot control for confounding factors, leading to some impact on the conclusions. For example, due to different molecular subtypes and physical conditions, the chemotherapy regimens used by these patients may vary. The effectiveness of different chemotherapy regimens are variants, which may affect the evaluation of treatment efficacy. Secondly, small sample size may lead to insufficient statistical power. There is not enough sample size to conduct subgroup analysis to exclude the influence of confounding factors and explore more possible outcomes. Thirdly, long term follow-up is insufficient to assess the prognosis of these patients, leading to the inability to explore the prediction value of SII and PNI on DFS or OS. Therefore, large-sample prospective and multicenter trials with sufficient clinicopathological and survival data are needed to support our findings.

## Conclusions

In conclusion, breast cancer patients with low ΔPNI, high ΔSII, and high ΔPNI-ΔSII scores had better neoadjuvant chemotherapeutic effect. The ΔPNI-ΔSII score is a convenient and useful marker, which is a promising predictor of therapeutic effect after neoadjuvant chemotherapy for breast cancer.

## Data Availability

The raw data supporting the conclusions of this article will be made available by the authors, without undue reservation.
